# Pooled retrospective analysis of 70 mg erenumab in episodic and chronic migraine: a two tertiary headache centers experience during clinical practice

**DOI:** 10.1007/s13760-021-01770-7

**Published:** 2021-08-18

**Authors:** P. Storch, P. Burow, B. Möller, T. Kraya, S. Heintz, N. Politz, S. Naegel

**Affiliations:** 1 Headache Center Jena, Department of Neurology, University Hospital Jena, Am Klinikum 1, 07747 Jena, Germany; 2 Headache Center Halle, Department of Neurology, University Hospital Halle, Ernst-Grube-Str. 40, 06120 Halle (Saale), Germany; 3 Department of Neurology, Hospital St. Georg, Delitzscher Str. 141, 04129 Leipzig, Germany

**Keywords:** Migraine, Prophylaxis, Erenumab, CGRP antibody, Real-life data, Monoclonal antibody

## Abstract

Erenumab is a monoclonal antibody, targeted against the calcitonin gene-related peptide (CGRP) receptor. Clinical studies have demonstrated prophylactic efficacy in both episodic (EM) and chronic migraine (CM). The aim of the present study is to evaluate the efficacy of treatment in tertiary headache centers under real-life conditions. In a retrospective analysis, the period of 3 months before and after initiation of erenumab therapy was compared. Relevant parameters (headache days, headache intensity, headache duration, acute medication, previous prophylaxis treatments) were collected from medical charts of all migraine patients (*N* = 82) who started treatment with erenumab between November 1st 2018 and May 1st 2019 at two tertiary headache centers in Germany. The sample included 68 female (82.9%) and 14 male patients aged between 22 and 78 years (mean 51.1 years, SD 10.5 years). Of these patients, 57.3% met the criteria for CM and 56.9% overused acute medication. Under therapy with erenumab, a significant reduction of headache days was observed from the first month on. The effect was most pronounced in the third month with a decrease in monthly headache days from 16.6 to 11.6 days (*p* < 0.001). There was also a significant reduction in reported headache intensity (*p* = 0.004) and average duration of headache attacks (*p* = 0.016). The 50% responder rate in patients with CM was lower in the first month compared to EM but then increased similarly to EM. Patients with medication overuse (MO) also responded to the therapy. There was a reduction in medication overuse from 57% at baseline to 29% after therapy (*p* = 0.011). Overall, a positive result of treatment with erenumab can be shown in a highly selected sample with severely affected migraine patients and a refractory course prior to treatment. This re-confirms the clinical trial data also for this highly selected group.

## Introduction

Migraine is a primary headache disorder with complex etiology, characterized by episodic headache and associated vegetative symptoms. With a prevalence of approx. 18%, migraine is of significant epidemiologic importance [1]. Besides frequently used medical acute therapy, headache prevention plays an important role in the treatment of migraines. In addition to non-pharmaceutic interventions, medications are important treatment options. These have so far mainly been several betablockers, flunarizine, anticonvulsants, and tricyclic-antidepressants, although the physiological mechanism of action of these drugs on the headache symptoms is largely unknown [2]. Often, treatment with these substances is discontinued due to undesirable side effects, insufficient efficacy, or contraindications [3, 4].

The new substance class of calcitonin gene-related peptide (CGRP) antibodies and CGRP receptor antibodies comprises the first drugs developed for migraine prophylaxis based on the pathophysiological knowledge of migraine. Erenumab is a fully humanized monoclonal antibody against CGRP and was the first approved substance for CGRP-based treatment in Germany based on its efficacy in chronic (CM) as well as episodic migraine (EM) [5–7]. Due to the significantly higher costs, reimbursement for CGRP-based therapies in Germany is limited to cases that are refractory to first-line prophylactics (beta-blocker, flunarizine, topiramate, amitriptyline, and for CM also onabotulinumtoxin A) or where these therapies were associated with intolerable side effects or were contraindicated.

Although the LIBERTY trial is often used to illustrate efficacy in therapy-refractory cases, it should be noted that the inclusion criteria of this study only allowed between two and four failed preventive treatments [6]. Therapy-refractory patients with more than four failed preventive treatments were not investigated in clinical trials. Therefore, studies are needed to clarify to which extent the data can be transferred to the currently mainly treated patients.

The Central German Headache Center Jena is a certified headache center (level 3) of the German Migraine and Headache Society (DMKG).

We present early real-world therapy data on the efficacy of erenumab in highly therapy refractory migraine patients.

## Methods

For retrospective analysis of the therapy, relevant documents of 82 patients treated with erenumab at the Headache Centers Jena and Halle were evaluated. In the Headache Center Jena about 1500 patients with migraines are treated every year. Erenumab was used here since 2018 for prophylactic migraine therapy. The second study site (Halle) treats about 1500 headache patients per year, the majority of them migraine patients. Erenumab here also has been used since 2018 for prophylactic migraine treatment.

The period considered for analysis included 3 months before and after initiation of erenumab therapy.

The diagnosis was made according to the current diagnostic criteria of the International classification of headache (ICHD 3) [8]. In line with the German approval of erenumab, patients had to have at least 4 migraine day per month and—due to the regulation of reimbursement—most of the patients had previously failed at least 5 (EM) or 6 (CM) preventive treatments (discontinuation due to side effects, contraindication or insufficient effect despite adequate dosage and therapy time).

The treatment regimen was the same at both sites. Since only 70 mg erenumab were initially available in Germany, all patients included in this analysis were treated exclusively with 70 mg within the observation period. A prerequisite for the initiation of therapy and inclusion was the documentation of headache days over 3 months prior to initiation of therapy (= baseline = BL).

As the primary endpoint, the average reduction in headache days from the 3-month BL compared to the 3-month treatment period was predefined.

The following secondary endpoints were additionally analyzed:

- Fifty percent responder rate regarding headache days (3-month BL vs. 3-month treatment period).
- Change in average headache intensity (0 = light, 1 = medium, 2 = severe; 3-month BL vs. 3-month treatment period).
- Average duration of headache episodes regardless of the intake of acute medication (0 = less than 6 h; 1 = 7 to 12 h, 2 = longer than 12 h; 3-month BL vs. 3-month treatment period).
- Frequency of acute medication intake (3-month BL vs. 3-month treatment period).
- Effectiveness of acute medication (0 = none; 1 = little, 2 = good; 3-month BL vs. 3-month treatment period).
- Medication overuse (MO) frequency (3-month BL vs. 3-month treatment period).
- Subgroup analysis (patients with vs. without MO) regarding the reduction of headache days and 50% responder rates.

Additionally, following parameters were descriptively evaluated:

- Number of previous attempts of drug-based migraine prophylaxis as well as a number of therapy discontinuations due to intolerance, ineffectiveness and/or contraindication.
- Number/percentage of patients with pain medication overuse (MO) at the start of therapy with erenumab (≥ 10 days of acute medication per month in each of the 3 months before initiation of erenumab therapy).
- Number of study discontinuations.

Data sources at each headache center consisted of headache diaries kept by patients and documentation performed by clinic staff (e.g., related to pre-existing diseases, prior medication, and headache-related medication). Because the patients did not all use the same headache diary, not all data were equally evaluable [e.g., headache intensity according to numeric rating scale (0–10)]. Therefore, only data that could be validly collected in all calendars were evaluated. The target data were initially identified by the treating neurologists in Jena and Halle. Subsequently, the data were entered into a prepared data mask by a research associate, prepared for statistical analysis and evaluated descriptively and inference-statistically. SPSS 25 (International Business Machines Corporation, Armonk, New York, USA) was used for analysis. The test procedures used are indicated in the results section of the respective results. Figures were generated using SigmaPlot 12.0 (Systat Software inc.; San Jose, CA, USA).

The local ethics committees of the Friedrich-Schiller University Jena and the Martin-Luther University Halle-Wittenberg independently approved the analysis. Subsequently, the anonymized data were pooled and analyzed.

## Results

### Cohort description

In total, data from 82 patients (68 female, 14 male) aged between 22 and 78 years (*M* = 51.12, SD = 10.66) were analyzed. At baseline (BL), datasets were complete in all 82 patients with respect to headache days. Data on headache days in individual months were missing for the first month in three patients, for month 2 in 23 patients, and for month 3 in eight patients. No patient discontinued treatment before the end of the observation period (3 months after BL), e.g. due to adverse effects or lack of treatment effect.

The baseline average number of headache days per month was 16.6 (range 5–30, SD = 6.86). Of the subjects investigated, 14.6% fulfilled the criteria for low-frequency EM (1–7 migraine days per month), 28% fulfilled the criteria for high-frequency EM (8–14 migraine days per month), and 57.3% fulfilled the criteria for CM (≥ 15 headache days per month) at BL.

For 76 patients, sufficient datasets were obtained to assess whether there was MO. 41 (53.9%) patients had MO in the 3 months before the start of erenumab therapy. Overuse was defined as the use of acute medication on ≥ 10 days per month for each of the 3 months prior to treatment.

Prior to initiation of erenumab therapy, an average of 4.49 (SD = 1.33) medical attempts were made to prevent migraines. Prophylactic medication was discontinued most frequently due to insufficient efficacy (average 2.61, SD 1.43) followed by intolerance (average 1.51, SD 1.29) and contraindications (average 0.89, SD 1.01). 59.8% of the total cohort (49/82) received at least one injection of onabotulinum toxin A. Detailed information on the frequency of previous preventive attempts is given in Table 1.

**Table 1:** Frequency of failed migraine preventives sorted by respective reasons

	Number of failed preventives					
	0	1	2	3	4	> 4
Lack of effectivity (*n* = 81)	7.4%	14.8%	28.5%	16.0%	25.9%	7.4%
Lack of tolerability (*n* = 81)	27.2%	29.6%	16.0%	19.8%	7.4%	0.0%
Contraindication (*n* = 80)	48.7%	21.3%	22.5%	7.5%	0.0%	0.0%

Depiction of frequencies (%) in relation to the number of preparations that were ineffective, intolerable, or contraindicated

### Comparison between study sites

In the Headache Center Jena 47 patients with a mean age of 49.28 (SD 9.20) and in Halle 35 patients with a mean age of 53.60 (SD 12.06) were recruited. The patients did not differ significantly regarding gender distribution, number of headache days at BL, the proportion of patients with CM and proportion of patients with MO at BL (*t* test).

## Effectiveness of therapy

### Influence of erenumab on the number of headache days within the first 3 months of treatment

Following treatment initiation, a significant reduction in headache days was observed. In the total cohort, an average reduction in headache days from the 3-month BL compared to the 3-month treatment period of 4.97 (SD 4.42, *n* = 82, *p* < 0.001) was observed. For 53 patients, monthly datasets are available from the BL and patient's control appointments. In this group, the mean reduction in headache days was 4.81 (SD 4.35, *p* < 0.001).

**Fig. 1:**
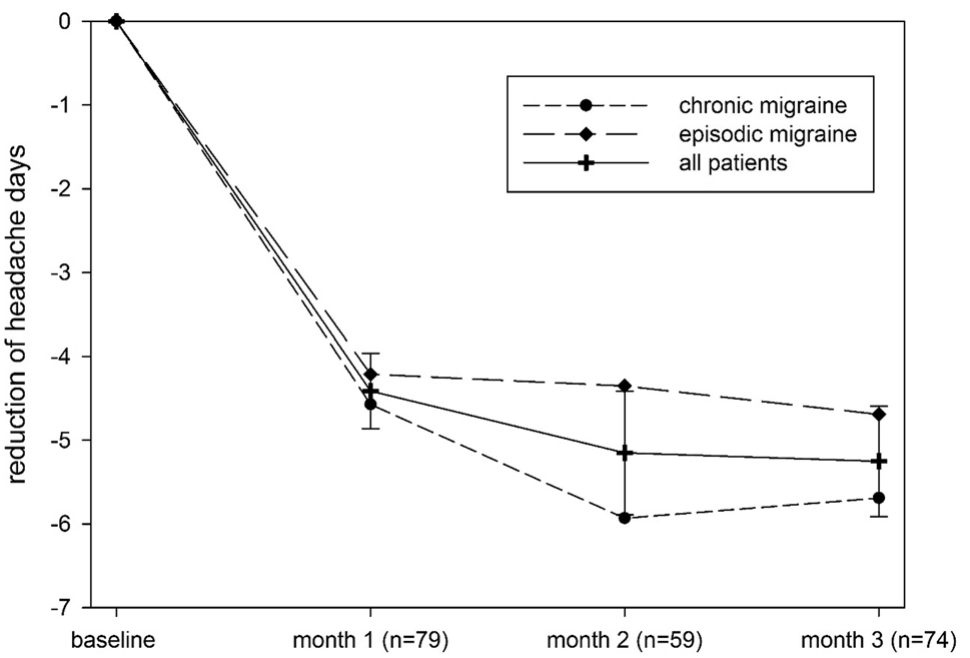
Reduction of headache days under erenumab (70 mg) therapy. Reduction of headache days for each month after initiation of erenumab treatment compared to mean BL. Numbers of patients are given as not all data were available

Efficacy was demonstrated from the first month of treatment onwards with a reduction of 4.41 headache days (SD 3.97, *n* = 79, *p* < 0.001) compared to BL. The effect further increased in the second month (reduction of headache days: 5.15, SD 5.65; *n* = 59; *p* < 0.001) and remained stable in the third month (reduction of headache days: 5.24, SD 5.64, *n* = 74, *p* < 0.001) (Fig. 1). The reduction was significant for both, CM and EM (*p* ≤ 0.001 for all 3 months compared to BL, paired *t* test; 1st month EM: 4.21 CM: 4.57; 2nd month EM: 4.35 CM: 5.93; 3rd month EM: 4.69 CM: 5.69).

**Fig. 2:**
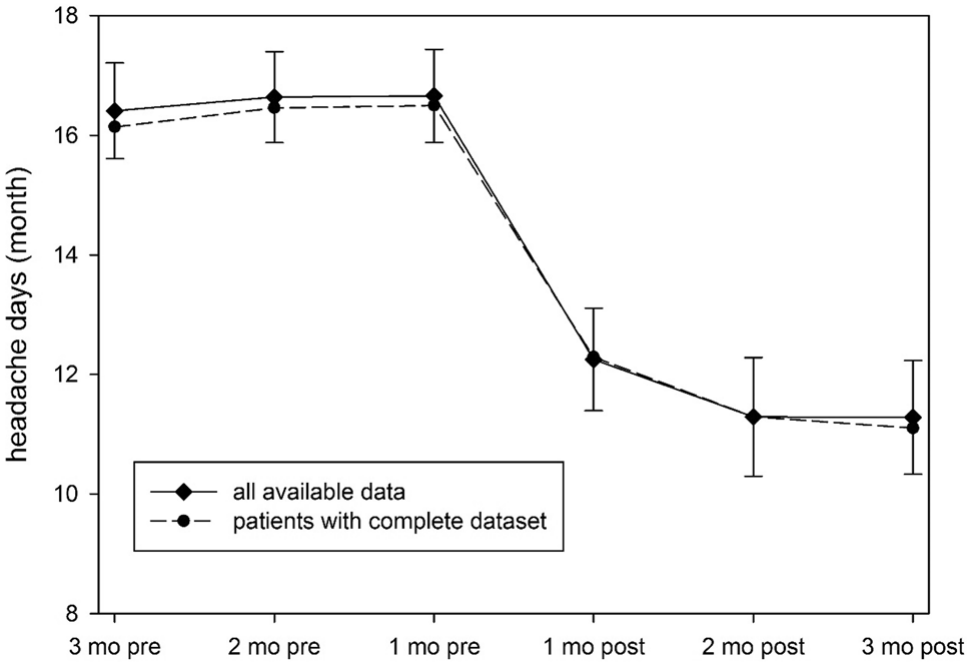
Course of headache days during the six-month observation period. In addition to the reported findings of the manuscript, the graph illustrates the immediate effect with significant reduction of headache days in the total cohort comparing the first month before (1 mo pre) and the first month after (1 mo post) treatment initiation with 70 mg erenumab

In the total cohort, a significant reduction of headache days was observed between the month before and the first month after treatment with erenumab (Fig. 2, *n* = 79, *p* < 0.001, paired *t* test).

### 50%-responder rate within and after a 3-month therapy with erenumab

**Fig. 3:**
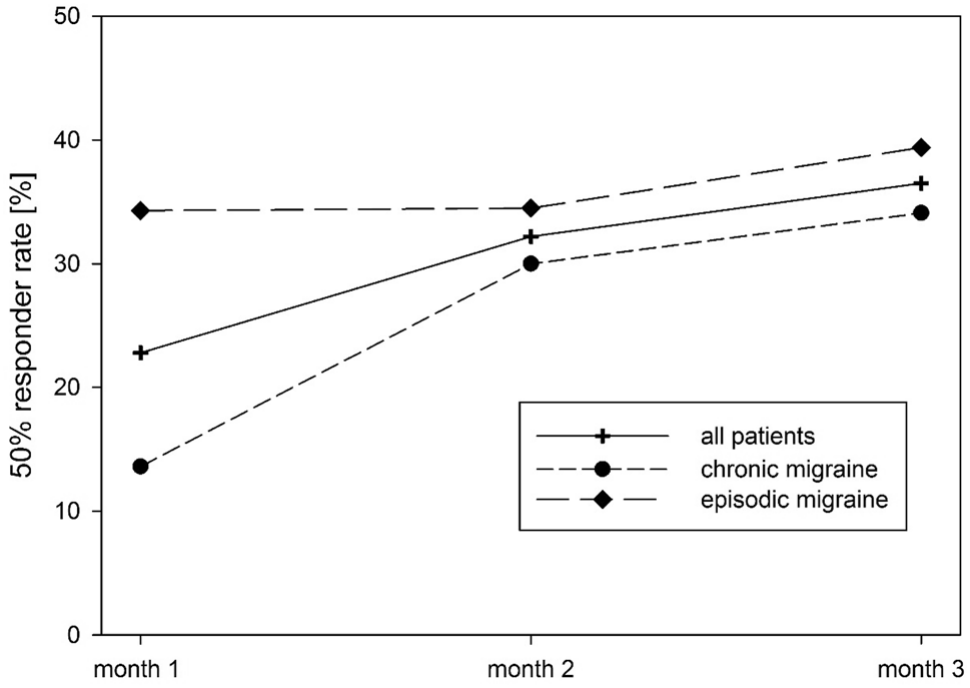
Development of 50% responder rate over the 3-month treatment period with 70 mg erenumab. Comparison of mean BL to headache days of every month, differentiating all migraine, episodic and chronic course. For month 1 the 50% responder rate was significantly different between patients with episodic and chronic migraine (see text)

Similar results were obtained for the total cohort with respect to the 50% responder rate in comparison to the 3-month BL (Fig. 3). For the first month, a 50% reduction was found in 23.1% (18 out of 79) of patients. The proportion with a 50% reduction of headache days further increased during the second (32.2% of patients, 19/59) and the third month (36.5% of patients, 27/74). Averaged over 3 months, the 50% responder rate was 26.9% (14/52). In patients with CM, the initial 50% responder rate was lower (first month 13.6%, 6/44, second month 30%, 9/30, third month 34.1%, 14/41), whereas patients with EM responded more quickly with respect to this parameter (first month 34.3%; second month 34.5%, third month 39.4%). However, the 50%-responder rate significantly distinguished between patients with EM and CM only in the first month after treatment with erenumab (*p* = 0.029, *n* = 79, Fisher’s exact test). A center effect could not be found for the 50% responder rate for any of the 3 months (month 1: *p* = 0.295, *n* = 79; month 2: *p* = 0.098, *n* = 54; month 3: *p* = 0.187, *n* = 74; Fisher’s exact Test).

In 24.3% of the patients, a maximum reduction of 10% in the number of headache days was observed in the third month after therapy compared to the mean BL. 17.6% of patients experienced an increase in headache days in this interval.

### Influence of erenumab on headache intensity

Datasets regarding headache intensity within the observed interval (6 months) were complete for 53 patients. There was a significant reduction in headache intensity when comparing the average BL period to the average treatment period. The self-reported mean intensity before treatment was 1.34 (SD 0.50) on a three-stage scale (0 = light, 1 = medium, 2 = severe). In the 3 months following treatment, this value decreased to 1.28 (SD 0.52, *p* = 0.02, paired *t* test).

For the averaged datasets of all patients, 78 datasets could be used showing similar results. The self-reported mean intensity before treatment was initially 1.44 (SD 0.51). In the 3 months following treatment, the mean intensity decreased to 1.31 (SD 0.55, *n* = 78, *p* = 0.004, paired *t* test).

### Impact of erenumab on the average attack-duration

Data from 30 patients could be used to evaluate the attack-duration. There was a significant reduction in the average duration. While a three-stage scale (0 = < 6 h, 1 = 7–12 h, 2 = > 12 h) showed an average attack length of 1.05 (SD 0.54) in the 3 months prior to the first injection, this was reduced to 0.93 (SD 0.56, *p* = 0.021, paired *t* test) in the 3 months after the first injection.

### Treatment influence on acute medication intake

For the evaluation of acute medication intake frequency datasets of 62 patients were available. Comparing baseline and therapy period a significant reduction of acute medication was observed. In the 3 months prior to therapy initiation, acute medication was used on 42.6% (SD 18.8) of the days. In the 3 months therapy period, the number of days with acute medication usage decreased to 29.2% (SD 18.1%) of the days (*p* < 0.001, paired *t* test).

### Influence of erenumab therapy on the effectiveness of acute medication

Regarding effectiveness (0 = no effect, 1 = little effect, 2 = good effect) of acute treatment, a significant difference between the baseline (mean 1.59, SD 0.42) and therapy period (mean 1.72, SD 0.35) was observed (*p* = 0.006, *n* = 66, paired *t* test).

### Impact of erenumab influence on present MO

Of all patients with sufficient datasets, 56.9% (33/58) met the criteria for MO in the 3 months before therapy initiation. This proportion declined 3 months after treatment with erenumab to 29.3% (17/58, *p* = 0.011).

### Influence of MO on therapy response

Patients with MO had a higher BL headache burden (19.97 days, SD 6.59, *n* = 41) than patients without overuse (12.87 days, SD 5.44, *n* = 36; *p* < 0.001, *t* test). After initiation of erenumab, headache days decreased significantly in both groups in all months compared to the mean BL (Table 2, *p* ≤ 0.001 for all months, paired *t* test). Although MO patients numerically responded better to erenumab treatment, this failed statistical significance (example 3rd month, MO vs. no MO: *p* = 0.203, *t* test). There was no significant difference in the 50% responder rates for all months compared to the median BL comparing patients with and without overuse (month 1: *p* = 0.587; month 2: *p* = 1000; month 3: *p* = 0.806, Fisher’s exact test). The results are summarized in Table 2.

**Table 2:** Efficacy of erenumab in patients with and without pain medication overuse (MO)

	Month 1		Month 2		Month 3	
	MO	No MO	MO	No MO	MO	No MO
Reduction of headache days (vs. mean BL)	4.68 ± 4.24, *n* = 41	3.88 ± 3.67, *n* = 35	6.88 ± 6.25, *n* = 27	3.11 ± 4.55, *n* = 29	5.86 ± 6.75, *n* = 36	4.1 ± 4.18, *n* = 33
50% responder rates	20% (8/41)	26% (9/35)	30% (8/27)	31% (9/29)	33% (12/36)	36% (12/33)

Comparative presentation of monthly headache day reduction and 50% responder rates in patients with and without medication overuse during therapy with erenumab. The numbers in parentheses indicate the available datasets

## Discussion

This study shows the effectiveness of 70 mg erenumab treatment in previously therapy-refractory migraine patients under real-life conditions at two independent headache centers.

A positive therapy effect could be illustrated on several target parameters. We found a significant reduction of headache days in all three treatment months. Regarding the 50% responder rates, we found a delayed response in patients with CM in the first month compared to EM. This confirms the data from a Phase II study and supports the common practice of assessing the therapeutic effect of the antibody especially in patients with chronic migraine only after 3 months [9, 10].

In addition to a reduction in the number of headache days, a 3-month therapy reduced the average intensity and duration of a headache attack. The consideration of pain intensity and duration provides important clues regarding the reduction of the individual burden in the context of migraines. In addition, erenumab treatment decreased the rate of MO by almost half in the observed population, although a drug holiday was not performed prior therapy. This is in line with the previously published Phase II trial of erenumab in CM, where efficacy in patients with migraine and medication overuse headache (MOH) was shown [7]. The efficacy of the acute medication increased significantly within the study in the 3 months after the first injection. This might reflect a reduced attack intensity. A direct effect of erenumab should also be discussed.

This study expands and confirms real-world data of studies investigating therapy effects of erenumab [11–16]. It should be noted that the results are not directly comparable as there were different compositions of study groups (regarding the proportion of patients with CM) and different doses of erenumab. Recently, two retrospective studies were published adding real-world experience with erenumab in tertiary headache centers in Germany [14, 16]. Regarding reduction of monthly headache days and 50% responder rates, the results of these studies could now be reproduced. Non-responders were described with a similar proportion [14]. Both studies showed a reduction of acute medication intake following erenumab, which is corroborated by our data. Only little data exist comparing the time course of the treatment effect in EM and CM. An Italian study by Ornello et al. found an increasing 50% responder rate over the first 3 months of erenumab therapy in a group almost exclusively composed of patients with CM [13]. This is also supported by our data showing significant differences in the early response of CM vs. EM. This underlines that especially CM patients need long enough evaluation periods before none-response is assumed.

Our data also provide information regarding the therapeutic effect of erenumab in patients with MOH. The 50% responder rate and reduction of monthly headache days are as good as in patients without MOH. This is in line with a previous study [12]. These results suggest that patients with MOH benefit from erenumab even without performing a withdrawal of acute medication. However, we were not able to confirm the results of the first Italian real-life data study presenting very high responder rates, possibly due to attrition-bias [17].

Limitations of the study mainly comprise missing data values and the retrospective study design. Data sets for individual parameters were not complete for individual months (e.g. 2nd month). The analyses per month showed congruent results and differed only slightly from the overall cohort and therefore appear to be transferable to the latter. As no study dropouts occurred, we do not see any risk of overestimating the therapeutic effect in our cohort. During antibody therapy, we did not analyze additionally used drugs or the use of non-drug treatment. The influence of these is unlikely, as in the clinical routine workup of both centers no additional therapies are started simultaneously. Regarding headache load, we limited the analysis to headache days and did not include migraine days for example, as patients’ differentiation of migraine- and headache-days is often unreliable and therefore by some authors is even interpreted as a continuum [18]. Accordingly, headache days may better reflect the actual burden in the target population.

Further research is needed to replicate the findings in a larger, prospective study design.

## Data Availability

The datasets analyzed during the current study are available from the corresponding author on reasonable request.
